# Pigmented fungiform papillae of the tongue in a Japanese child

**DOI:** 10.1002/ccr3.2830

**Published:** 2020-04-12

**Authors:** Yuki Sugiyama, Katsuhiko Hayashi, Takeshi Takayama

**Affiliations:** ^1^ Department of Dentistry The Jikei University School of Medicine Tokyo Japan

**Keywords:** fungiform papillae, pigmentation, tongue

## Abstract

Pigmented fungiform papillae of the tongue (PFPT) does not require invasive investigation and treatment. However, if the patient requests treatment for aesthetic reasons, and the pigmentation is focally distributed, an excisional biopsy can be chosen for both diagnosis and treatment.

## Graphical abstract

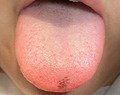

## 1 INTRODUCTION

We report a case of pigmented fungiform papillae of the tongue in a 9‐year‐old Japanese girl. Invasive treatment was not necessary for the lesion, but an excisional biopsy was performed under local anesthesia at her parents’ request, for aesthetic reasons. No recurrence was found at the 3‐year follow‐up.

Papillae on the surface of the tongue may be filiform, fungiform, or circumvallate. Fungiform papillae are mushroom‐shaped projections that contain taste buds, are pink or red in color, and located on the lateral or dorsal surfaces, and mainly on the tip, of the tongue.^1^, ^2^ Pigmented fungiform papillae of the tongue (PFPT) are characterized by circumscribed hyperpigmentation localized to the fungiform papillae. These lesions are asymptomatic and nonprogressive, and generally develop in late childhood.^1^, ^3^ PFPT is more common in dark‐skinned adults and children; the prevalence is reportedly low among people of Asian and European descent.^3^ Although many dermatologists rarely inspect patients’ oral cavities during routine examinations, most cases of PFPT were previously reported by dermatologists.^4^ Oral surgeons and dentists are more likely to find PFPT and should be knowledgeable regarding the pathophysiology of these lesions. We report the clinical course and microscopic findings of PFPT in a Japanese child, which was treated by excisional biopsy.

## 2 CASE REPORT

**FIGURE 1:**
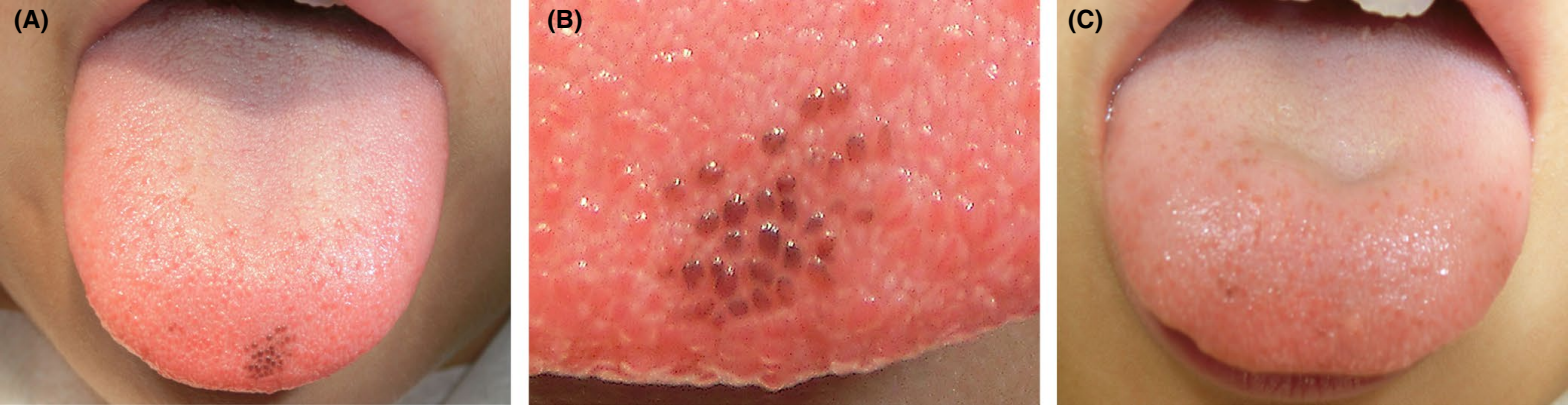
Macroscopic findings at (A) low‐power magnification and (B) high‐power magnification at the first medical examination. Darkly pigmented fungiform papillae are seen on the anterior aspect of the dorsum of the tongue. Macroscopic finding at 3 years after excisional biopsy (C) indicates no recurrence of the lesion

A 9‐year‐old Japanese girl was referred to our outpatient clinic for the diagnosis of a patchy‐colored region on her tongue that had been noticed by her parents for the past month. At the first visit, intraoral examination showed asymptomatic darkly pigmented fungiform papillae on the anterior aspect of the dorsum of the tongue (Figure 1A). Macroscopic examination revealed approximately 30 fungiform papillae with pigmentation within the lesion (Figure 1B). Similar pigmentation was absent from other parts of the dorsum of the tongue and oral mucosa. All blood test results, including a hemogram and adrenal cortex function testing, were within normal limits. The remainder of the physical examination was normal. A diagnosis of PFPT was made, and a 6‐month follow‐up was recommended. However, the patient's parents strongly requested excision of the pigmented area for aesthetic reasons and to obtain a definitive diagnosis. An excisional biopsy was performed under local anesthesia. The wound healing after the biopsy has been uneventful, and the patient did not complain of paresthesia or dysmotility. No recurrence was found at the 3‐year follow‐up (Figure 1C).

**FIGURE 2:**
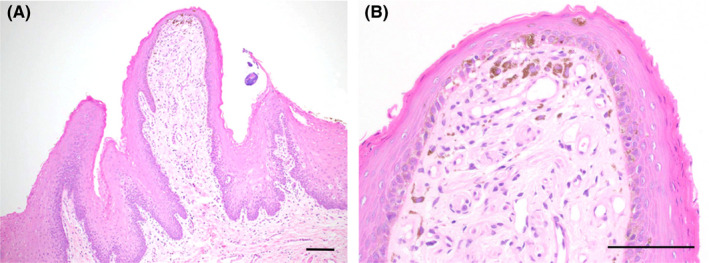
Histopathological findings at (A) low‐power magnification and (B) high‐power magnification with hematoxylin and eosin staining. Melanocytes and melanophages are seen mainly near the tip of the fungiform papillae. Melanocytes are present along the basal cell layer of the epithelium, and melanophages are seen within the connective tissue core under the epithelium. Scale bar = 100 µm

Histopathological examination showed that the fungiform papillae consisted of stratified squamous epithelium overlying loose connective tissue. Melanocytes containing brown melanin granules were present along the basal cell layer of the epithelium, and several melanophages containing melanin granules were observed within the connective tissue core under the epithelium. These melanocytes and melanophages were seen mainly near the tip of the fungiform papillae (Figure 2A,B).

## 3 DISCUSSION

Pigmented fungiform papillae of the tongue was first described in 1905 and was suggested to be associated with endemic anemia.^5^ After many cases were subsequently reported, PFPT was considered a common variant of the oral hyperpigmentation that is seen in individuals with intensely pigmented skin.^1^ Holzwanger et al^1^ examined 300 random individuals and reported that among patients of African descent, 30% of men and 25% of women had some hyperpigmentation of the fungiform papillae. In contrast, among people of Asian descent, the prevalence of PFPT was reportedly very low,^3^ and Tan et al^6^ reported that the prevalence was 0.4% in Chinese population. Additionally, Chessa et al^4^ recently reviewed 193 cases of PFPT among 23 articles in the English‐language literature and found that 60% (116 cases) of PFPT occurred in individuals of African descent, and 35% (68 cases) occurred in individuals of Asian descent. Further study of the precise onset rate among different races is needed.

Holzwanger et al^1^ classified PFPT into three distinct clinical types: (a) well‐defined hyperpigmented macules involving all fungiform papillae and located on the anterolateral surface or tip of the tongue; (b) hyperpigmentation involving 3‐7 fungiform papillae and randomly distributed on the dorsal surface of the tongue; and (c) hyperpigmentation of all fungiform papillae on the dorsal surface of the tongue.^1^ PFPT is most commonly of the second clinical type and is distributed on the dorsal lingual surface; the first pattern is reported mainly in children.^4^ In our patient, the pigmentation pattern was of the first type, similar to patients in previous reports.^4^

In PFPT, hyperpigmentation is secondary to an accumulation of melanin in macrophages in the fibrous connective tissue under the epithelium. Histopathologically, PFPT shows prominent melanophages in the connective tissue without abnormal melanogenesis in the epithelium or inflammation in the lamina propria.^1^ These melanophages contain abundant melanin, which is extruded from the melanocytes in the basal cell layer of the epithelium. In our patient, melanocytes and melanophages were seen mainly near the tip of the fungiform papillae, and this distribution pattern is considered to accentuate the coloration of this condition.

The differential diagnoses of PFPT are pernicious anemia, hemochromatosis, Peutz‐Jeghers syndrome, Addison's disease, Laugier‐Hunziker syndrome, melanocytic nevi, melanoma, black hairy tongue, and amalgam tattoo.^1^, ^2^ These conditions can be distinguished from PFPT according to the patient's clinical findings, clinical images, and the appropriate clinical inspection for each disease. When asymptomatic macular pigmentation of the portions of the tongue containing taste buds is observed, PFPT must be preferentially considered as a diagnosis. Invasive investigation and treatment are not necessary, and no effective treatment has been reported. However, if the patient requests treatment for aesthetic reasons, and the pigmentation is focally distributed, an excisional biopsy can be chosen for both diagnosis and treatment.

## CONFLICT OF INTEREST

None declared.

## AUTHOR CONTRIBUTIONS

YS: collected clinical data and conceived and produced the first draft of the manuscript. KH: contributed to patient evaluation, diagnosis, and treatment, and critically revised the manuscript. TT: drafted the manuscript and supervised the final draft.
